# Therapeutic Effect of Xuebijing, a Traditional Chinese Medicine Injection, on Rheumatoid Arthritis

**DOI:** 10.1155/2020/2710782

**Published:** 2020-01-28

**Authors:** Shutong Li, Hongxing Wang, Qinbao Sun, Bin Liu, Xiaotian Chang

**Affiliations:** ^1^Medical Research Center of the Hospital Affiliated of Qingdao University, Wutaishan Road 1677, Qingdao, Shandong 266000, China; ^2^Department of Medicine, Pingdingshan University, Chongwen Road, Pingdingshan, Henan 467000, China; ^3^Rizhao Ren'ai Hospital, Weihai Road 69, Rizhao, Shandong, China; ^4^Rheumatic Disease Department of the Hospital Affiliated of Qingdao University, Wutaishan Road 1677, Qingdao, Shandong 266000, China; ^5^Qingdao Engineering Technology Center for Major Disease Markers, Wutaishan Road 1677, Qingdao, Shandong 266000, China

## Abstract

**Background:**

Traditional Chinese medicine considers that rheumatoid arthritis (RA) is caused by blood stasis, heat, and toxins. Xuebijing (XBJ), a traditional Chinese medicine compound injection, activates blood circulation to dissipate blood stasis, eliminating pathogenic heat from the blood and degrading toxins. XBJ was approved by the China FDA to treat for many years. This study examined the potential therapeutic effects of XBJ on RA and rat collagen-induced arthritis (CIA).

**Methods:**

XBJ was cultured with the synovial fluid (SF) of RA patients. XBJ was also injected into CIA rats. Changes in Treg and Th17 cell levels in the peripheral blood (PB), SF, and spleen and changes in Th1/Th2 and cytokine levels in PB were detected using flow cytometry. Four RA patients were treated using XBJ based on Chinese medical theory and Chinese medicine indications.

**Results:**

Following culture with XBJ, the proportion of Treg cells (*P*=0.007) was significantly increased in RA SF, while the Th1/Th2 ratio remained unchanged. After XBJ treatment, CIA in rats was significantly relieved (*P*=0.007) was significantly increased in RA SF, while the Th1/Th2 ratio remained unchanged. After XBJ treatment, CIA in rats was significantly relieved (*β*, IL-6, IL-17A, IFN-*γ*, and TNF-*α* decreased (*P*=0.007) was significantly increased in RA SF, while the Th1/Th2 ratio remained unchanged. After XBJ treatment, CIA in rats was significantly relieved (*P*=0.007) was significantly increased in RA SF, while the Th1/Th2 ratio remained unchanged. After XBJ treatment, CIA in rats was significantly relieved (*P*=0.007) was significantly increased in RA SF, while the Th1/Th2 ratio remained unchanged. After XBJ treatment, CIA in rats was significantly relieved (*P*=0.007) was significantly increased in RA SF, while the Th1/Th2 ratio remained unchanged. After XBJ treatment, CIA in rats was significantly relieved (*P*=0.007) was significantly increased in RA SF, while the Th1/Th2 ratio remained unchanged. After XBJ treatment, CIA in rats was significantly relieved (

**Conclusion:**

XBJ can restore the immune balance to treat RA and CIA. Therefore, XBJ could be a potential therapeutic drug for RA.

## 1. Introduction

Rheumatoid arthritis (RA) is a common autoimmune disease [1, 2]. Tumor necrosis factor *α* (TNF-*α*) and interleukin-6 (IL-6) levels are significantly elevated in RA, which stimulates osteoclast formation and the destruction of the articular cartilage and bone and simultaneously induces the production of other inflammatory cytokines [3].

T regulatory (Treg) cells are differentiated from CD4+ T cells [4]. They can inhibit macrophage production of TNF-*α* and IL-6 inflammatory cytokines, directly contact dendritic cells, and inhibit the expression of major histocompatibility complex (MHC) II molecules to reduce their antigen presentation abilities [5–7]. Therefore, Treg cells play an anti-inflammatory role in RA. The functions of T helper 17 (Th17) cells are opposite to those of Treg cells. Th17 cells secrete IL-17A to induce TNF-*α* and IL-6 expression and mediate inflammatory cell infiltration. This causes articular cartilage and bone injury, thereby playing a proinflammatory role in RA [8]. The balance between Treg cells and Th17 cells has been considered an important cause of RA development [9]. Additionally, Th1 cells are mainly characterized by IFN-*γ* secretion and can mediate cellular immunity, whereas Th2 cells mainly secrete IL-4 and mediate humoral immunity [10]. An imbalanced Th1/Th2 cell ratio also contributes to RA development, while reduction of the Th1/Th2 ratio has therapeutic effects on RA [11, 12].

Xuebijing (XBJ) is a compound injection of five Chinese herbs: radix paeoniae rubra, rhizoma chuanxiong, *Salvia miltiorrhiza*, flos carthami, and *Angelica sinensis* [13–15]. The active ingredients include paeoniflorin, ferulic acid, danshensu, and hydroxysafflor yellow [16]. XBJ activates blood circulation to dissipate blood stasis, eliminating pathogenic heat from the blood and degrading toxins. Clinically, XBJ is mainly used for the treatment of sepsis, infection-induced systemic inflammatory response syndrome, and multiple organ dysfunction syndrome [17]. It can also be used to treat the organ dysfunction stage of multiple organ dysfunction syndrome. It has been shown that XBJ can relieve inflammatory responses through regulation of the immune balance and inhibition of excessive TNF-*α* and IL-6 release [18]. XBJ can stimulate an increase in the number of Treg cells in mice with sepsis and can reduce the levels of TNF-*α* and IL-6 in the serum to increase the survival rate of mice with sepsis [19].

Traditional Chinese medicine hypothesizes that RA is caused by blood stasis, heat, and toxins. Thus, this study aimed to determine whether XBJ had therapeutic effects on RA and to investigate the treatment mechanism. This study established a collagen-induced arthritis (CIA) rat model. We examined the therapeutic effect of XBJ on CIA by injecting XBJ through the tail vein. This study also used XBJ to culture synovial fluid (SF) from RA patients. We observed changes in Treg cell and Th17 cell proportions and the Th1/Th2 ratio. Additionally, this study treated 4 RA patients with XBJ to validate the effects of XBJ on the treatment of RA. We conducted this treatment based on the traditional Chinese medicine therapeutic concept of activating blood circulation to dissipate blood stasis, eliminating pathogenic heat from the blood and degrading toxins of RA, which are covered by the indication of the medicine. This study investigates whether XBJ treatment could restore immune balance in RA by regulating the ratio of Treg/Th17 or Th1/Th2 cells.

## 2. Materials and Methods

### 2.1. Sample Collection

SF samples from RA patients (*n* = 11) were collected after articular cavity puncture. The samples were obtained from the Department of Orthopedic Surgery in The Affiliated Hospital of Qingdao University.  Table 1 describes clinical information of the sample donors. This study also recruited 4 RA patients for XBJ treatment in the Shandong Rizhao Ren'ai Hospital. Peripheral blood (PB) samples were collected from the patients before and after treatment.  Table 2 describes the clinical information of those 4 RA patients. All enrolled RA patients conformed to the RA classification standard released by the American College of Rheumatology (ACR) in 1987. This study was approved by the Ethics Committee of The Affiliated Hospital of Qingdao University and the Ethics Committee of Rizhao Ren'ai Hospital. All enrolled patients signed an informed consent form.

### 2.2. Culture of SF and XBJ

SF samples were pretreated in hyaluronidase at 37°C for 30 min. The lymphocyte suspension was isolated using lymphocyte separation solution (TBDscience, China). After centrifugation, 5 × 10^5^ cells were resuspended in Dulbecco's modified Eagle's medium (DMEM, HyClone, USA) containing 10% fetal calf serum (FCS, Biological Industries, Israel), inoculated into 24-well plates, and incubated with 10 *μ*l of XBJ (CHASE SUN, China) for 48 h. The total cell suspension was collected, anti-human-CD4-FITC antibody (BioLegend, USA) and anti-human-CD25-PE antibody (BioLegend) were added, and the mixture was incubated at 4°C in the dark for 30 min. The proportion of Treg cells in the SF before and after XBJ stimulation was measured using a flow cytometer (ACEA, USA). Next, the cell suspension was centrifuged at 1000 ×g and 4°C for 15 min. The supernatant was used to measure changes in the secretion levels of IL-4 and IFN-*γ* using the human Th1/Th2 cytokine detection reagent kit (Cell-Genebio, China). The mean fluorescence intensity (MFI) was detected using a flow cytometer. Th1 cells are characterized by IFN-*γ* secretion, while Th2 cells mainly secrete IL-4. Based on the obtained MFI values of IFN-*γ* and IL-4, the Th1/Th2 ratio was calculated.

### 2.3. Establishment of Collagen-Induced Arthritis (CIA) Rats and XBJ Treatment

A total of 60 Sprague Dawley (SD) rats (6-week-old, male) were purchased from Pengyue Experimental Animal Breeding Co., Ltd., in Jinan, China. The animals were randomly divided into the normal control (NC) group, the CIA group, and the XBJ treatment group (20 animals/group). Bovine type II collagen (Chondrex, USA) and complete Freund's adjuvant (Sigma, USA) were mixed and fully emulsified. The first immunization was performed by intracutaneous injection into the tail root of the rats. After 1 week, bovine type II collagen and incomplete Freund's adjuvant were mixed and fully emulsified. During booster immunization, the XBJ treatment group received an injection of XBJ (5 ml/kg) through the tail vein once every 3 days for a total of 6 injections. The NC and CIA groups received an injection of an equal volume of PBS at the same time. Rats were euthanized with lethal doses of ketamine and xylazine.

### 2.4. Evaluation of General Conditions of Rats

At the start of XBJ treatment, the swelling degree of the ankle joint of the foot was measured with a vernier caliper every 3 days. The inflammation curve of changes in the degree of joint swelling over time was plotted.

### 2.5. Histopathological Examination of Rat Joints

After 20 days of XBJ treatment, the animals were anesthetized by intraperitoneal injection of 3% pentobarbital at 30 mg/kg. The areas approximately 0.5 cm around the knee joint were collected. Tissues were fixed in 4% paraformaldehyde (Solarbio, China) and embedded into paraffin.

### 2.6. Detection of Rat Lymphocyte Subsets

On day 8 and day 20 of XBJ treatment, blood samples were collected from the inferior vena cava. Meanwhile, the animals were immobilized at the left lateral position to expose the right knee joint, and PBS was injected into the articular cavity for lavage. The lavage fluids were recovered for future use. The spleen was completely removed, placed in PBS, minced, and filtered to obtain a single-cell suspension. The PB, spleen single-cell suspension, and articular cavity lavage fluid samples were treated to isolate a lymphocyte suspension using rat lymphocyte separation solution (TBDscience). The cell suspension was then mixed with the anti-rat-CD4-FITC antibody (BioLegend) and anti-rat-CD25-PE antibody (BioLegend) and incubated at 4°C in the dark for 30 min. Changes in the Treg cell proportion were detected using a flow cytometer.

Before Th17 cell detection was performed, phorbol ester, ionomycin, and monensin (MultiSciences, China) were added to the lymphocyte suspension, and the mixture was incubated at 37°C for 4 h. After treatment, the single cell suspension was mixed with anti-rat-CD4-FITC and incubated at 4°C in the dark for 30 min. The fixation solution was added and incubated at room temperature in the dark for 15 min according to the manufacturer's protocol of the intracellular staining reagent kit (BioLegend). Next, the membrane lysis solution was added, and the sample was centrifuged at 350 ×g for 5 min. After two washes, the cells were resuspended in membrane lysis solution, and anti-rat-IL-17A-PE (eBioscience, USA) was added. Changes in Th17 cells were detected using a flow cytometer.

Changes in the secretion levels of IL-4 and IFN-*γ* in PB samples were detected using the rat Th1/Th2 cytokine detection reagent kit (BioLegend). The Th1/Th2 ratio was calculated according to the MFI values of IFN-*γ* and IL-4.

### 2.7. Detection of Inflammatory Cytokines in PB of Rats

After 20 days of XBJ treatment, blood samples were collected from the inferior vena cava. The expression levels of cytokines in the PB were detected using a rat inflammatory cytokine flow cytometry assay kit. The surfaces of microspheres with different fluorescence intensities were coated with specific antibodies against rat IL-1*β*, IL-6, IL-17A, IFN-*γ*, and TNF-*α*. The serum sample, microspheres, and buffer were mixed thoroughly and incubated at room temperature in the dark for 2 h. After washing, the biotinylated detection antibody and avidin-coupled PE antibody were added. The MFI values of the above inflammatory cytokines were detected using a flow cytometer.

### 2.8. Treatment of RA Patients Using XBJ

Patients did not receive hormone and immunosuppressant treatment or biotherapy within at least 2 months of the study. Four RA patients received daily intravenous injection of 50 ml of XBJ according to the drug instructions and indication for 7 consecutive days. During the treatment, drugs that might influence the treatment evaluation, such as disease-modifying antirheumatic drugs (DMARDs), hormone drugs, and nonsteroidal anti-inflammatory drugs (NSAIDs), were not used. PB samples of patients before and after XBJ treatment were collected for detection of routine clinical indicators, including the erythrocyte sedimentation rate (ESR), C-reactive protein (CRP), and rheumatoid factor (RF) levels. The disease activity was analyzed using the disease activity score-28 (DAS28).

### 2.9. Detection of Lymphocyte Subsets in RA Patients

PB samples from patients before and after XBJ treatment were collected. Lymphocytes were isolated and suspended using human peripheral blood lymphocyte isolation solution (TBDscience). Detection of Treg cells and an analysis of the Th1/Th2 ratio were performed according to the methods for detection of cells in the SF of RA patients. Detection of Th17 cells was performed according to the instructions of the human Th17 staining reagent kit (MultiSciences). Before detection, the phorbol ester/ionomycin/monensin mixture solution was added to the lymphocyte suspension and incubated at 37°C. After 4 h, the single-cell suspension was mixed with the anti-human-CD3-FITC antibody and anti-human-CD8-PerCP antibody and incubated at 4°C in the dark for 15 min. The fixation and membrane lysis solution A of the kit was added, and the sample was incubated at room temperature in the dark for 15 min. Next, the fixation and membrane lysis solution B of the kit and anti-human-IL-17A-PE antibody were added and incubated at 4°C in the dark for 15 min. Th17 cell quantities before and after XBJ treatment were detected using a flow cytometer.

### 2.10. Statistical Treatment

SPSS 17.0 software was used for the normality and variance homogeneity tests. Data conformed to the examination standard were expressed as $\bar{x}\pm s$. The significance test among multiple groups was performed using ANOVA of repeated measurement design and one-way ANOVA. Pairwise comparison was performed using the LSD method or the Tamhane method. Data that did not conform to normality and variance homogeneity were expressed as *M* (*Q*_R_). The comparison between groups was performed using the nonparametric rank-sum test. *P* < 0.05 indicates statistical significance between groups.

## 3. Results

### 3.1. Effect of XBJ on SF of RA Patients

**Figure 1:**
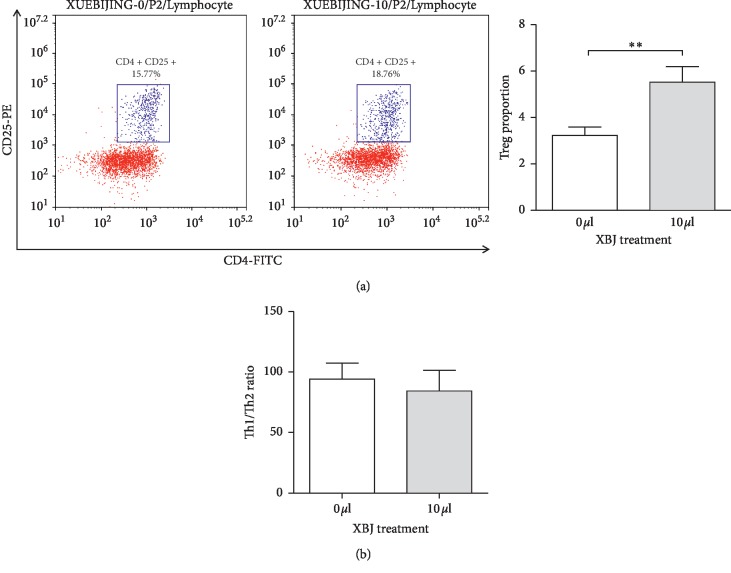
Detection of the effect of XBJ on SF (synovial fluid) from RA patients using flow cytometry. (a) Changes in the Treg cell population in SF after XBJ stimulation and statistical analyses. (b) Changes in the Th1/Th2 ratio in SF after XBJ stimulation. ^*∗*^*P* < 0.05 and ^*∗∗*^*P* < 0.01.

After culturing the clinically collected SF samples from RA patients with XBJ for 48 h, the proportion of Treg cells was significantly increased (*P*=0.007) compared to that in the group treated with PBS as a control (Figure 1(a)). Although the Th1/Th2 ratio in SF showed a decreasing trend after the XBJ stimulation, the difference did not have statistical significance (Figure 1(b)).

### 3.2. Effect of XBJ on CIA in Rats

**Figure 2:**
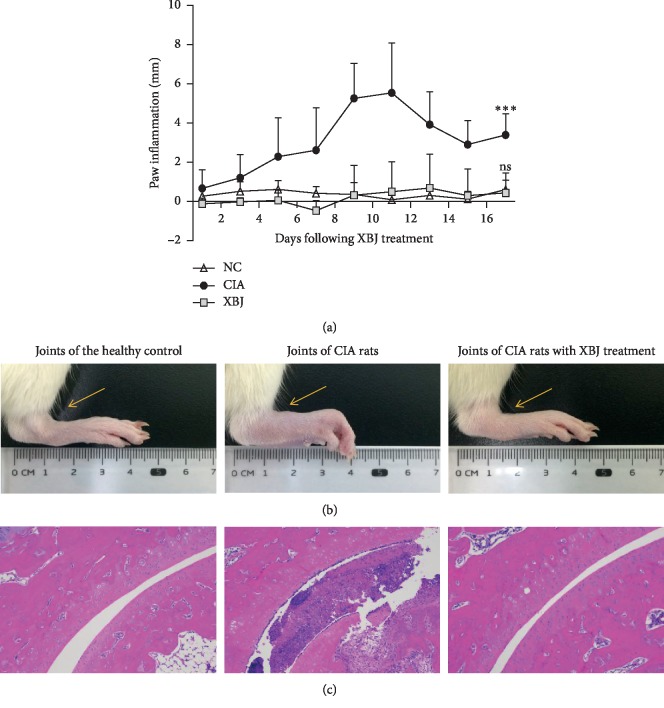
Effect of XBJ on arthritis in CIA rats. (a) Inflammation curve based on changes in the degree of joint swelling over time. (b) Appearance of rat joints. (c) HE staining of rat joint tissues. ^*∗∗∗*^*P* < 0.001. ns: not significant.

By day 8 of XBJ treatment, according to the inflammation curve, the degree of swelling in the ankle joints in the CIA group (5.27 ± 1.78 mm) was much higher than that of swelling in the NC group (0.35 ± 0.6 mm), and the difference was statistically significant (*P* < 0.001). The degree of swelling in the XBJ group (0.32 ± 1.53 mm) was significantly lower than that in the CIA group (*P* < 0.001) and was not significantly different from that in the NC group. On day 20, although arthritis in the CIA group decreased, the degree of swelling in the ankle joints in the CIA group (39 ± 1.10 mm) was still significantly greater than that of swelling in the NC group (0.60 ± 0.48 mm) and XBJ treatment group (0.44 ± 1.01 mm) (*P* < 0.001). At this time point, there was no significant difference between the XBJ treatment group and the NC group (Figure 2(a)). When compared to those in the NC group, the ankles and toes in the CIA group had inflammatory swelling and redness. The swollen joints had a certain degree of movement disorder, and the lower limbs could not touch the ground during walking. In the XBJ group, redness and swelling were not obvious, and activity was not limited (Figure 2(b)). These rats were sacrificed 20 days after XBJ injection. HE staining showed necrotic and exfoliated tissue masses in the articular cavity of rats that were treated with collagen II, and there was a large amount of inflammatory cell infiltration. There was partial defect of articular cartilage, and the cartilage surface had a monolayer of attached inflammatory cells. There were obvious synovial hyperplasia, increased capillaries, and a large amount of inflammatory cell infiltration in the CIA rats. In the XBJ group, the joint structure was intact, the articular cavity was clean, the bilateral cartilages were smooth, and there was no obvious synovial hyperplasia or pannus formation. Only a small amount of inflammatory cell infiltration was observed in the connective tissues (Figure 2(c)).

**Figure 3:**
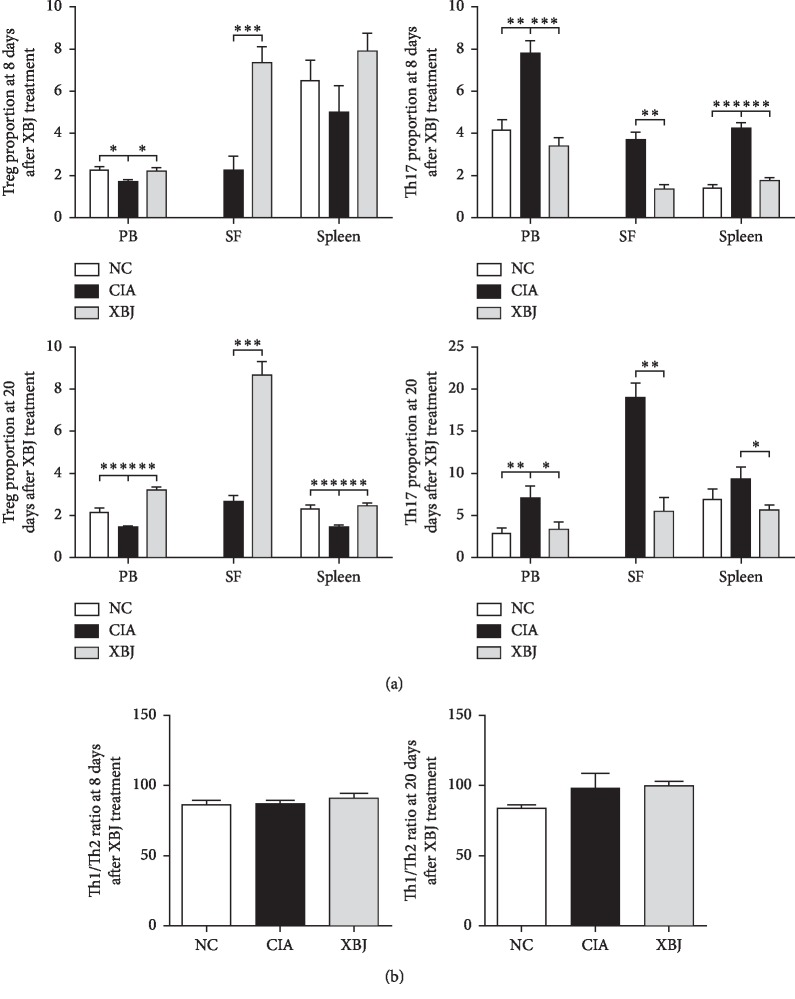
Effect of XBJ treatment on the immune balance of CIA rats. (a) Changes in the proportions of Treg and Th17 cells in the PB (peripheral blood), SF (synovial fluid), and spleen of the rats on day 8 and day 20 after XBJ treatment. (b) Changes in the Th1/Th2 ratio in PB of the rats on day 8 and day 20 after XBJ treatment. ^*∗*^*P* < 0.05, ^*∗∗*^*P* < 0.01, and ^*∗∗∗*^*P* < 0.001.

The rats were sacrificed on day 8 and day 20 after XBJ treatment. The levels of Treg and Th17 cells and the ratio of Th1/Th2 cells were measured. On day 8 of XBJ treatment, the proportion of Treg cells in the PB of CIA rats significantly decreased (*P*=0.012), while the proportion of Th17 cells significantly increased (*P*=0.001) compared to that in the NC group. Meanwhile, the proportion of Treg cells in the PB significantly increased after XBJ treatment (*P*=0.021), while the proportion of Th17 cells significantly decreased compared to that in the CIA group without treatment (*P*=0.01). Similarly, the proportion of Treg cells in SF in the XBJ treatment group was significantly higher than the proportion of these cells in the CIA group (*P* < 0.001), and the proportion of Th17 cells was significantly lower compared to that in the CIA group (*P*=0.001). At this time point, the proportion of Treg cells did not differ significantly in the spleen among the three groups, although the proportion showed an upward trend in the XBJ treatment group. The proportion of Th17 cells in the spleen was greater in the CIA group than in the other groups (*P* < 0.001 and *P* < 0.001). On day 20 of XBJ injection, the proportion of Treg cells in lymphocytes in the PB and spleens of CIA rats without XBJ treatment decreased (*P*=0.002 and *P* < 0.001), and the proportion of Th17 cells in the PB increased (*P*=0.005) compared to that in the NC group. After XBJ treatment, the proportion of Treg cells in the PB and spleen significantly increased (*P* < 0.001 and *P* < 0.001), while the proportion of Th17 cells significantly decreased (*P*=0.013 and *P*=0.029) compared to that in the CIA group without XBJ treatment (*P*=0.01). The proportion of Treg cells in the XBJ treatment group SF was significantly higher than the proportion in the CIA group (*P* < 0.001), and the proportion of Th17 cells was significantly lower than that in the CIA group (*P*=0.001) (Figure 3(a)). On day 8 and day 20, changes in the Th1/Th2 ratio in the PB among the 3 groups were not significantly different (Figure 3(b)).

**Figure 4:**
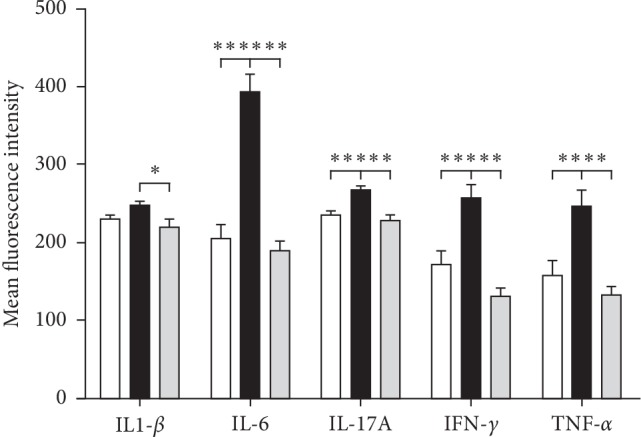
Effect of XBJ treatment on peripheral blood cytokines of CIA rats. ^*∗*^*P* < 0.05, ^*∗∗*^*P* < 0.01, and ^*∗∗∗*^*P* < 0.001.

On day 20 after XBJ treatment, the levels of IL-6, IL-17A, IFN-*γ*, and TNF-*α* in the PB of rats in the CIA group were all higher than those in the NC group, and the differences were statistically significant (*P* < 0.001, *P*=0.002, *P*=0.001, and *P*=0.003, respectively). The levels of IL-1*β*, IL-6, IL-17A, IFN-*γ*, and TNF-*α* in the XBJ group all significantly decreased compared to those in the CIA group (*P*=0.031, *P* < 0.001, *P* < 0.001, *P* < 0.001, and *P*=0.001, respectively) (Figure 4).

### 3.3. Effect of XBJ on RA Patients

**Figure 5:**
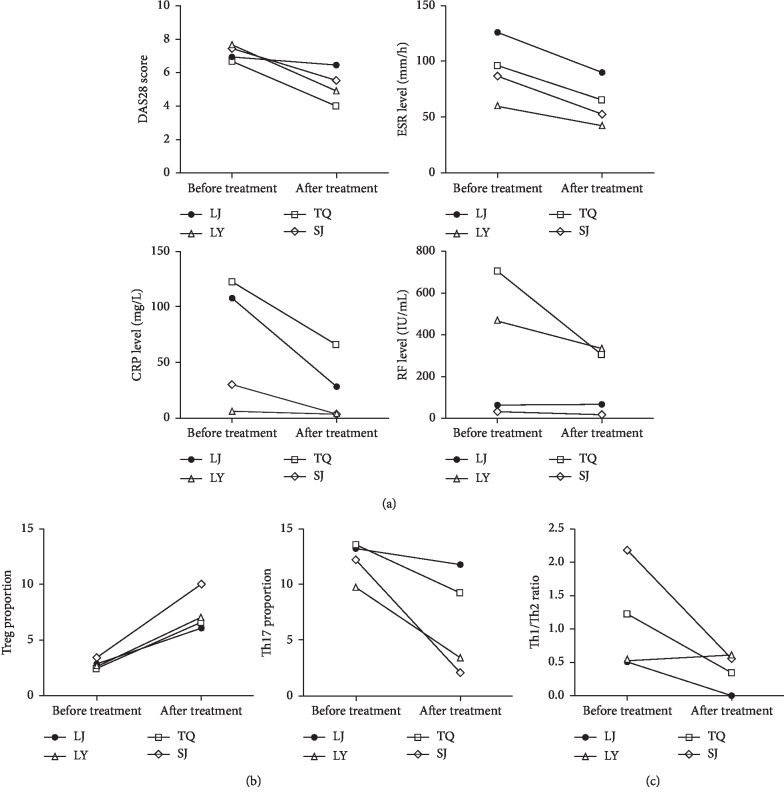
Effect of XBJ treatment on RA patients. (a) Changes in clinical indicators of the patients. (b) Changes in Treg and Th17 cell levels in patient PB. (c) Changes in the Th1/Th2 ratio in patient PB.

This study used XBJ to treat 4 RA patients according to the drug's instructions and indications as well as Chinese medical theory for 7 consecutive days. Compared to the scores before treatment, the DAS28 scores significantly decreased in all 4 patients after the treatment, with corresponding decreases in the ESR level. The CRP level decreased in 3 of the 4 patients, and the RF level significantly decreased in 2 of the 4 patients (Figure 5(a)). The Treg cell proportion significantly increased in all 4 patients after the treatment, whereas the Th17 cell proportion decreased in all patients (Figure 5(b)). The ratio of Th1/Th2 decreased in 3 patients and remained unchanged in 1 patient (Figure 5(c)).

## 4. Discussion

The animal study results indicated that the severity of arthritis in rats after XBJ treatment was significantly decreased compared to that in the CIA group. Histopathological sections also showed that the articular cartilage in the rats after XBJ treatment was intact, and there was no obvious synovial hyperplasia or pannus formation, indicating significant alleviation of arthritis in the CIA rats following XBJ treatment. This study also used XBJ injection to treat 4 RA patients according to the drug's instructions and indications. After 7 consecutive days of injection, the DAS28 scores, ESR level, and CRP level significantly decreased, and the RF level significantly decreased in 2 of the 4 patients. IL-6 and TNF-*α* are at the center of the RA cytokine network [3, 20]. IL-1*β* also plays a proinflammatory role in RA and participates in Th17 cell differentiation [21, 22]. Our results showed that the levels of IL-1*β*, IL-6, IL-17A, and TNF-*α* were higher in the CIA group than in the NC group and were lower in the XBJ treatment group. Our results suggested that XBJ may treat CIA by reducing inflammatory cytokine levels. These results all confirmed that XBJ had a significant therapeutic effect on RA and CIA.

The *in vitro* experiments performed in this study showed that XBJ stimulation could significantly increase the proportion of Treg cells in the SF of RA patients. On day 8 and day 20 of XBJ treatment, the Treg cells in the PB, spleen, and SF of rats significantly increased, while the Th17 cells decreased compared to those in the CIA group without treatment. In addition, the proportion of Treg cells increased and the proportion of Th17 cells decreased in the 4 RA patients after XBJ treatment, consistent with the animal experiment results. The above results suggest that XBJ might exert its therapeutic effect on RA by increasing Treg cell levels and decreasing the proportion of Th17 cells.

Although XBJ stimulation did not alter the Th1/Th2 ratio in the SF of RA patients and the animal experiment results showed that the Th1/Th2 ratio in the PB of the rats did not significantly change, a comparison among the 4 RA patients showed that the Th1/Th2 ratio decreased in 3 of the patients. These results suggest that XBJ may also regulate the Th1/Th2 balance in human RA.

Sepsis rats generated by cecal ligation puncture had significantly reduced TNF-*α* and IL-6 levels after XBJ treatment [6]. XBJ stimulated Treg cell differentiation and inhibited Th17 cell differentiation *in vitro*, thus increasing the number of Treg cells that secreted IL-10 in sepsis rats and reducing neutrophil infiltration in the lung and kidney [7]. These reports are similar to our observations.

The development of RA is closely associated with immune system disorders [23]. Under normal physiological conditions, the biological functions of Treg and Th17 cells antagonize each other, forming a balance that maintains normal immune responses and prevents the development of autoimmune diseases [24]. Some scholars hypothesize that the basis of RA development is the imbalance between Treg and Th17 cells and their secreted cytokines [25]. The Th17 cell-secreted IL-17A cytokine significantly increases in the PB of RA patients [26, 27]. In the active stage of the disease, the imbalance between Treg and Th17 cells is even more evident. Clinical studies have indicated that the Treg cell level in the PB of patients negatively correlates with the DAS28 score of RA, while the Th17 cell level positively correlates with this score [28, 29]. Additionally, the imbalance of Th1/Th2 is also closely associated with the development of RA. It has been shown that Th1 cell differentiation increases and Th2 cell differentiation decreases in RA patients [30]. A reduction of the Th1/Th2 ratio can help inhibit inflammatory responses and treat RA [31]. Based on the current studies of XBJ, we hypothesized that XBJ might restore the immune cell balance to play a therapeutic role in RA through increasing the amount of Treg cells, reducing Th17 cells, or even influencing the Th1/Th2 ratio.

## 5. Conclusions

The *in vivo* and *in vitro* experiments performed in this study all confirmed that XBJ has therapeutic effects on RA and CIA by elevating the proportion of Treg cells and reducing the proportion of Th17 cells. This investigation utilized immunological, animal, and clinical studies to support the application of XBJ as an RA therapeutic drug.

## Figures and Tables

**Table 1:** Clinical information of synovial fluid donors.

Patient	CL	ZZ	CY	ZY	YL	DM	DH	QJ	ZL	WJ	SC
Age (years)	58	62	65	50	70	52	48	28	44	65	76
Sex	F	F	F	F	F	F	F	F	F	F	M
Diagnosis	RA	RA	RA	RA	RA	RA	RA	RA	RA	RA	RA

F, female; M, male; RA, rheumatoid arthritis.

**Table 2:** Clinical information of patients receiving XBJ treatment.

Patient	LJ	LY	TQ	SJ
Age (years)	56	62	77	57
Sex	M	F	M	F
Diagnosis	RA	RA	RA	RA

M, male; F, female; RA, rheumatoid arthritis.

## Data Availability

The data used and/or investigated during the present study are available from the corresponding author upon reasonable request.

## Abbreviations

CIA: Collagen-induced arthritis
CRP: C-reactive protein
DAS28: Disease activity score-28
DMARDs: Disease-modifying antirheumatic drugs
ESR: Erythrocyte sedimentation rate
IL-6: Interleukin-6
MHC: Major histocompatibility complex
NSAIDs: Nonsteroidal anti-inflammatory drugs
PB: Peripheral blood
RA: Rheumatoid arthritis
RF: Rheumatoid factor
SF: Synovial fluid
TNF-*α*: Tumor necrosis factor *α*
Treg cells: T regulatory cells
Th17 cells: T helper 17 cells
XBJ: Xuebijing.
